# Exosome enriched serum enhances engineered ligament mechanics and collagen content with no additional benefit of resistance exercise

**DOI:** 10.1016/j.mbplus.2025.100181

**Published:** 2025-08-08

**Authors:** Kevin J.M. Paulussen, Christopher M.T. Hayden, Taylor Griffin, Keith Baar

**Affiliations:** a Department of Physiology and Membrane Biology, University of California, Davis, CA, USA; b Neurobiology, Physiology and Behavior, University of California, Davis, CA, USA

**Keywords:** Tendon, Muscle, Exercise, Ligament, Exosomes

## Abstract

Following resistance exercise, systemic changes foster improved functionality of tendons and ligaments. Post-exercise, muscle tissue releases exosomes that are thought to facilitate inter-tissue communication. To determine the potential role of exosomes in the exercise-induced adaptations of tendons and ligaments, we modified our engineered human ligament (EHL) model to work with exosome-enriched serum. Treatment of the EHLs with exosomes enriched from fetal bovine serum (fbEXO) resulted in enhanced ligament mechanics and increased collagen content in a dose–response fashion (maximum tensile load [MTL]: 10 %: 0.196 ± 0.138 N, 20 %: 0.278 ± 0.103 N, 40 %: 0.840 ± 0.092 N; r^2^ = 0.858, *P* < 0.0001; collagen content: 10 %: 1.073 ± 12.49 µg, 20 %: 86.43 ± 71.65 µg, 40 %: 145.7 ± 84.11 µg; r^2^ = 0.4735, *P* = 0.0046). After optimizing an exosome enriched feeding protocol using fbEXO, we confirmed that exosomes enriched from human serum (hsEXO) could sustain EHL function. Subsequently, twelve healthy, recreationally active volunteers (22 ± 3 y, 1,68 ± 0.10 m, 65.6 ± 27.8 kg; 6F/6M) performed a single bout of resistance exercise. Serum samples were collected prior to and 15 min post-exercise, and exosomes were enriched from these samples for treatment of EHLs. EHL function and collagen content did not differ when treated with hsEXO obtained at rest or post-resistance exercise (MTL: 1.30 ± 0.36 vs. 1.20 ± 0.36 N, *P* = 0.3950; collagen content: 424.6 ± 47.68 vs. 425.2 ± 44.46 µg, *P* = 0.9663). This model provides a novel way to determine the role of exosomes in connective tissue development and adaptation. The identification of circulating exercise factors that enhance tendon and ligament function remains to be fully elucidated.

## Highlights

• Exosomes enriched from fetal bovine serum can sustain engineered human ligament (EHL) function for up to 4 days in culture.
• Treating EHLs with exosome enriched from human serum results in mature ligaments with high tensile strength.
• Exosomes isolated after heavy resistance exercise had no greater effect on EHL mechanics or collagen content.
• This novel technique will allow researchers to determine the role of exosomes in connective tissue development and adaptation.

## Introduction

Musculoskeletal injuries are among the most common afflictions that prevent people performing activities of daily living, going to work, and engaging in regular physical activity. These issues affect an estimated 1.71 billion people affected worldwide [1]. Ligament and tendon injuries are the most common diagnosis, accounting for 45 % of all musculoskeletal injuries [2]. Currently, the only method for strengthening ligaments and tendons is exercise, with a supportive role for nutrition [[3], [4]]. Exercise can also induce systemic changes that facilitate the repair and adaptation of connective tissues that were not directly loaded [5]. Resistance exercise in particular has been suggested to enhance tendon size and mechanical properties [6,7,8], thereby improving athletic performance [9].

Adaptations in connective tissues induced by exercise stem predominantly from the increased stress or strain associated with the activity. For instance, tendons experience heightened mechanical tension during muscle contraction, which promotes enhanced collagen synthesis [10]. When repeated over time, the result is a larger tendon that is mechanically more robust [[11], [12],^13^]. Beyond the direct effect of mechanical load, exercise can stimulate the release of proteins and other signaling molecules from muscle and other tissues, termed exerkines [14]. Exerkines have been hypothesized to contribute to the systemic adaptation to exercise. Several mechanisms have been postulated for how these systemic factors contribute to connective tissue adaptations. For example, changes in the hormonal milieu (i.e., sex hormones, growth hormone, IGF-1) are known to affect connective tissue cells *in vitro* [15,16], and IGF-1 is required for load-induced increases in tendon size *in vivo* [17]. These data suggest that protein factors released from muscle may contribute to improved tendon function through muscle–tendon crosstalk [18]. However, results from our lab suggest that post-resistance exercise changes in growth hormone, IGF-1, or transforming growth factor (TGF) β1 do not explain the systemic increase in connective tissue mechanics and collagen content that occur after resistance exercise [5]. Therefore, the mechanisms underlying muscle–tendon crosstalk after resistance exercise remain to be elucidated.

Following exercise, muscle secretes not only proteins but also exosomes—small extracellular vesicles that originate from inward buds of the plasma membrane [19]. Exosomes contain not only proteins, but also nucleic acids (micro [miRNA], messenger [mRNA], and long non-coding [lnRNAs] RNAs as well as mitochondrial DNA [mtDNA]). The possibility that one or more of these exosomal cargoes could facilitate inter-tissue communication has been hypothesized [19,20]. However, whether exosomes play a role in the exercise-induced adaptations of tendons and ligaments remains to be elucidated.

This study aimed to investigate the role of exosomes in the exercise-induced adaptations of connective tissue. First, we determined whether exosome enrichment of serum could sustain human engineered ligament (EHL) collagen content and function. Second, we determined the amount of human exosome enriched serum that was needed to grow EHLs. Last, we compared the impact of human exosome-enriched serum from individuals at rest or post-resistance exercise on the mechanical properties and collagen content of EHLs. We hypothesized that exosomes enriched from serum collected after exercise would enhance EHL mechanics and collagen content better than those isolated from the same individuals at rest.

## Methods

### Participants, blood sampling, and exercise

Twelve healthy recreationally active young adults (22 ± 3 y, 1,68 ± 0.10 m, 65.6 ± 27.8 kg; 6F/6M) consented to a protocol that was approved by the Institutional Review Board at the University of California Davis and that was written in accordance with standards set by the *Declaration of Helsinki*. Blood was drawn from an antecubital vein at rest and 15 min after an acute bout of resistance exercise. A similar exercise protocol had been used previously in our lab to stimulate an endogenous hormone response and resulted in stronger more collagen dense EHLs [5]. In short, after a warm-up, participants completed a bout of lower body resistance exercise (Pendulum, Rogers Athletic, Farwell, MI). The resistance exercise consisted of five sets of a knee-dominant leg press (Pendulum Seated Squat Pro) with 1 min rest intervals between sets, three sets of lying hamstring curls (Pendulum Prone Leg Curl), and three sets of a hip-dominant leg press (Pendulum Hip Press). Blood samples were centrifuged (1500 g X 10 min) after clotting for at least an hour. The resulting serum was collected in a sterile environment, aliquoted, and frozen at −80 °C until used.

### Serum exosome enrichment

Extracellular vesicles sized 50–200 nm, comprising mainly exosomes, were enriched from human serum using a spin column-based procedure with affinity membrane binding according to manufacturer’s instructions (exoEasy, QIAGEN GmbH, Hilden, Germany). In short, serum was mixed 1:1 with a 2X binding buffer and added to a membrane affinity column to bind the exosomes to the membrane. After centrifugation, the flow-through was discarded and the membrane washed twice with a wash buffer to reduce non-specific material. An elution buffer was added to the column and centrifuged to collect the retained plasma exosomes. This process was repeated after which the solution was directly used for feeding engineered ligaments.

### Exosome enrichment confirmation

Additional blood from a single subject was drawn to confirm exosome enrichment. Exosome-enriched samples were prepared for transmission electron microscopy (TEM) by placing 20 µL of sample onto glow-discharged carbon-coated copper grids and negatively staining with uranyl acetate for 5 min. Samples were imaged by TEM (FEI Talos L120C, Thermo fisher, Waltham, MA, USA) and vesicle morphology and size were assessed to confirm the presence of exosome-like structures.

Presence of exosomal markers were quantified in both whole serum and exosome fractions via western blot analysis. Serum samples were diluted 1:50 in sucrose lysis buffer (1 M Tris HCl [pH 7.5], 1 M sucrose, 100 µM EDTA, 100 µM EGTA, 10 % NP40, and 1X protease inhibitor cocktail [cOmplete, Mini; Roche]). Exosome enriched eluates were evaporated under vacuum and reconstituted in 60 µL sucrose lysis buffer. After determination of protein concentration by a DC protein assay, samples were diluted in Laemmli sample buffer, boiled for 5 min at 100 °C, and sonicated for 5 s. Western blots were performed with 15 µL samples at 0.73 µg/µL. Samples were run on Bio-Rad SDS-PAGE 4–20 % Criterion TGX stain-free gels at constant voltage (200 V). Total protein within the gel was imaged following 1 min UV activation. Proteins were transferred to a nitrocellulose membrane and membranes were blocked for 30 min in 5 % skim milk in tris-buffered saline with 0.1 % Tween-20 (TBST), washed three times, and then incubated with primary antibodies at 4 °C overnight. Secondary antibodies conjugated to HRP were diluted 1:10,000 in TBST and membranes were incubated for 1 h at room temperature. Subsequently, membranes were briefly incubated with chemiluminescent HRP substrate (Millipore Immobilon) and imaged using the Bio-Rad ChemiDoc™ MP System. Image Lab 5.0 software (Bio-Rad) was used to detect protein bands. The primary antibodies used were HSP70 [Cell Signaling #4872], Flotillin-1 [Cell Signaling #18634], ALIX [Cell Signaling #92880], CD9 [Cell Signaling #13174], and CD63 [Cell Signaling #52090].

### Engineered ligament formation

Engineered ligaments were formed from primary cells obtained from the remnants of a human anterior cruciate ligament (hACL) as described previously [21]. Informed consent was obtained from the tissue donor according to a protocol approved by the Institutional Review Board at the University of California Davis. Briefly, the tissue remnant was sterilized in a 5 % antibiotic antimycotic solution (ABAM; Gibco, Thermo Fisher Scientific, Waltham, MA, USA) and subsequently digested for 16 h in a 0.1 % Type II Collagenase solution dissolved in Dulbecco’s modified Eagle’s medium (DMEM) containing 20 % fetal bovine serum and 1 % ABAM. After digestion, the resulting fibroblast population was plated onto 15 cm tissue culture plates and expanded in growth medium consisting of DMEM containing 10 % fetal bovine serum and 1 % penicillin. The cells were frozen in liquid nitrogen in aliquots containing DMEM, 20 % FBS and 10 % dimethyl sulfoxide (DMSO) until needed for experiments. Frozen cells were thawed and expanded by trypsinization to passage 4 prior to experiment use. At passage 4, 2.5 x 10^5^ cells were embedded in a fibrin gel on 35 mm plates containing two teardrop-shaped brushite (calcium phosphate) anchors placed 12 mm apart, used to mimic the bony ends of ligaments and to aid in mechanical testing. Over the next 4 days the cells contracted the fibrin into a ligament-like sinew between the two brushite anchors [22]. Constructs were fed every other day and maintained in feed medium consisting of growth medium supplemented with 5 ng/ml TGF-β1 (Peprotech, Rocky Hill, NJ, USA), 200 µM ascorbic acid and 50 µM proline unless otherwise noted.

### Development of ligament constructs treatment protocol

An optimal exosome enrichment and feeding protocol was developed utilizing exosomes isolated from FBS. First, ligament constructs were treated for 8 days with standard feed medium. For the remaining 6 days, constructs received media containing either complete FBS (10 %, positive control) or exosomes isolated from FBS (fbEXO) equivalent to 10 %, 20 %, or 40 % of the volume of feed media (exosomes from 200, 400, 800 µL of FBS for 2 mL of DMEM, respectively). TGF-β1 was omitted from any feed medium enriched with exosomes. The control feed with 10 % complete FBS contained TGF-β1, as established by previous protocols [21,23]. The primary objective of this experiment was to investigate whether exosome enriched media could support the growth and development of EHLs, determined by their mechanics and collagen content. We included a regular feed media control primarily to provide baseline context rather than for statistical comparison. From the exosome dose–response, the optimal dosage was selected to maximize construct mechanical properties and collagen content using the smallest possible amount of serum from each volunteer. Next, the constructs were fed 40 % fbEXO (exosomes enriched from 800 µL of serum equivalent to 40 % of the volume of feed media) for 2, 4, or 6 days to establish the minimal length of time needed to detect differences in EHL mechanics and collagen as a function of feeding. All engineered ligaments were cultured uniformly under standard media conditions containing TGFβ and FBS until day 8, 10, or 12, at which point exosome-enriched feeding commenced. Thus, each experimental condition inherently shares the same control (14 days under standard conditions). Finally, we used the established optimal dose and optimal time of treatment to determine whether treatment with exosomes enriched from human serum (hsEXO) obtained at rest led to viable constructs when compared to treatment according to our standard protocol (FBS).

### EHL human exosome treatment

Following the optimization experiments, EHLs were treated for 4 days with medium containing hsEXO isolated from a volume of serum equivalent to 30 % of the feed medium (i.e., each construct was fed with 2 mL of growth media that was supplemented with exosomes isolated from 600 µL human serum). The serum was obtained from participants in either a resting state or 15-min post-exercise. All exosome enriched feed media also contained 1 % penicillin, 200 µM ascorbic acid and 50 µM proline to permit collagen synthesis and export.

### Ligament construct tensile testing

On day 14, constructs were tensile tested as described previously [21]. In short, the length of each construct was measured using digital calipers and the width and thickness of each construct were determined by spectral-domain optical coherence tomography (OQ Labscope, Lumedica, Durham, NC) before the ligament anchors were placed into 3D printed grips attached to a single column tensile tester (Model 68SC-1, Instron, Norwood, MA) containing a 10 N load cell. The samples were submerged in 37 ˚ C saline and mechanically tested by loading to failure at a constant rate of 0.25 mm/s, following ten 0.075 N pre-conditioning cycles. From the tensile test, maximal load at failure was determined (MTL). Additionally, failure stress was calculated by normalizing MTL by the cross-sectional area and Young’s modulus was calculated as the maximal slope of the stress–strain curve.

### Collagen analysis

After mechanical testing, ligaments were removed from anchors and dried on a glass plate at 120 ˚ C for 30 min. Dry mass was determined and samples were left sealed at room temperature until processed for measurement of total collagen content. To determine collagen content, a hydroxyproline assay was performed as described previously [24]. In short, dried constructs were hydrolyzed in 200 µL of 6 N HCl at 120 ˚ C for two hours. Subsequently, samples were evaporated and reconstituted in 200 µL hydroxyproline buffer. Ten microliters of this solution was diluted to a final volume of 200 µL using hydroxyproline buffer. Chloramine-T solution (150 µL) was added and the samples were incubated for 20 min after which 150 µL aldehyde-perchloric acid (60 % 1-proponal, 5.8 % perchloric acid, and 1 M 4-(dimethylamino)benzaldehyde) was added for 15 min at 60˚ C. Samples (200 µL) were loaded in duplicate on a 96-well plate alongside hydroxyproline standards. Absorbance was read at 550 nm using an Epoch Microplate Spectrophotometer (BioTek Instruments Ltd, Winooski, VT, USA) and hydroxyproline content was calculated using a standard curve. Hydroxyproline was converted to collagen mass assuming that collagen contains 13.7 % hydroxyproline [25]. The collagen fraction was determined by dividing the collagen content by the mass of the tissue.

### Statistical analysis

For the experimental treatments utilizing human serum, a minimum of two replicates per condition per serum donor were used and the average mechanics and collagen content of the duplicates were used for all analyses. If the difference between the replicates was greater than 20 %, the experiment was re-run if sufficient serum remained. For all EHL experiments we randomized construct assignment across treatment groups to avoid position- or plate-specific biases.

Data were analyzed using a one-way ANOVA or student’s *t*-test using GraphPad Prism (Prism 10.4.0; Graphpad Software, San Diego, CA, USA). Tukey’s post hoc analysis was used to determine differences when the ANOVA indicated that significant differences existed. Linear regressions were performed separately, and linear fit was calculated using Prism. Statistical significance was set at *P* < 0.05. All data are presented as mean ± standard deviation.

## Results

### Biochemical and morphological validation of exosome enrichment from human serum

**Fig. 1:**
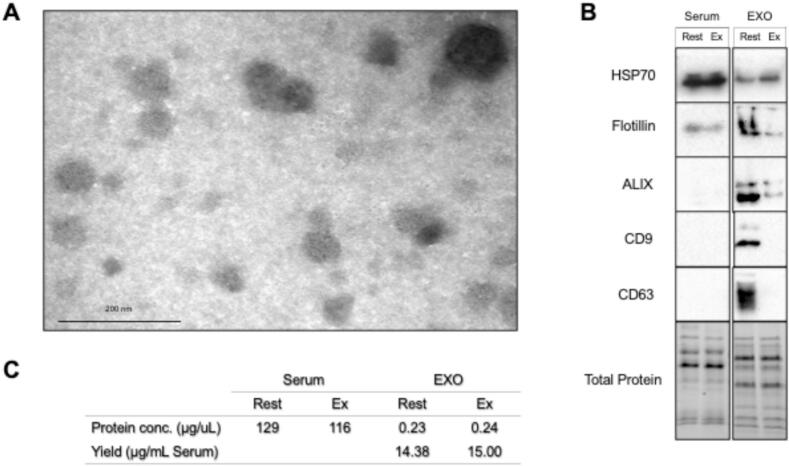
Confirmation of exosome enrichment from human serum under resting and post-exercise conditions. (**A**) Transmission electron micrograph of eluate containing enriched exosomes from human serum, showing characteristic exosome morphology and size (30–150 nm). Scale bar = 200 nm. (**B**) Western Blot analysis of known exosome markers. HSP70 and Flotillin were detected in both serum and exosome fractions, while ALIX, CD9, and CD63 were enriched in the exosome fraction and not detectable in crude serum. Total protein staining was used to confirm equal protein loading. (**C**) Quantification of total protein concentration in serum and exosome fractions by DC protein assay. Yield signifies µg protein recovered per mL of serum input, demonstrating comparable exosome recovery at rest and post-exercise.

Transmission electron microscopy revealed vesicular structures ranging from 30-150 nm with typical round to cup-shaped morphology, consistent with exosomes (Fig. 1**A**). Western blot analysis confirmed the presence of canonical exosome-associated proteins. HSP70 and Flotillin were detectable in both whole serum and the enriched exosome fraction. ALIX, CD9, and CD63 were only observed in the exosome fraction (Fig. 1**B**). Flotillin and ALIX showed lower concentrations in the post-exercise EXO samples when compared to the resting EXO samples. CD9 and CD63 were not detected in the post-exercise EXO samples, whereas clear bands were observed in the resting EXO samples. Protein concentrations were consistent between resting and post-exercise samples in both whole serum and exosome fractions. The enriched exosome fraction showed substantially lower protein concentrations (0.23 and 0.24 µg/µL) compared to the whole serum (129 and 116 µg/µL for resting and post-exercise samples, respectively. Fig. 1**C**). Together, these data confirm the enrichment of extracellular vesicles with exosome-like properties from human serum and support the use of this preparation for downstream treatment of our EHLs.

### fbEXO increases EHL mechanics and collagen content in a dose-dependent manner

**Fig. 2:**
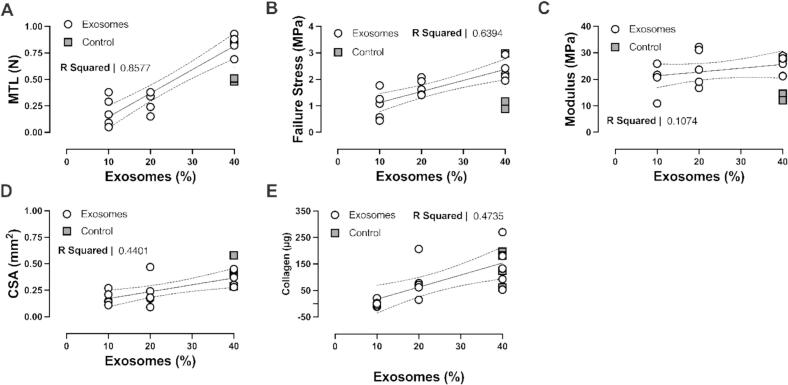
Dose-response relationship between FBS-derived exosome (fbEXO) concentration and engineered human ligament mechanical properties and collagen content. (**A)** Maximal tensile load (MTL), (**B**) failure stress, (**D**) cross sectional area (CSA), and (**E**) collagen content show a significant linear relationship with the amount of fbEXO. (**C**) Modulus does not change as a function of exosome concentration. Controls are fed with regular FBS as per our usual protocol and serve as a reference baseline to our routinely established ligament mechanics. Dotted lines represent 95% confidence interval.

Increasing the amount of exosomes enriched from FBS (fbEXO) within the media resulted in a dose-dependent increase in MTL (10 %: 0.196 ± 0.138 N, 20 %: 0.278 ± 0.103 N, 40 %: 0.840 ± 0.092 N; r^2^ = 0.858, *P* < 0.0001; Fig. 2A), failure stress (10 %: 1.024 ± 0.539 MPa, 20 %: 1.692 ± 0.302 MPa, 40 %: 2.326 ± 0.383 MPa; r^2^ = 0.639, *P* = 0.0003; Fig. 2B), area (10 %: 0.176 ± 0.064 mm^2^, 20 %: 0.230 ± 0.144 mm^2^, 40 %: 0.368 ± 0.078 mm^2^; r^2^ = 0.440, *P* = 0.0070; Fig. 2D), and collagen content (10 %: 1.073 ± 12.49 µg, 20 %: 86.43 ± 71.65 µg, 40 %: 145.7 ± 84.11 µg; r^2^ = 0.4735, *P* = 0.0046Fig. 2E). Modulus did not change as a function of exosome concentration (10 %: 20.14 ± 5.56 MPa, 20 %: 24.57 ± 6.95 MPa, 40 %: 25.02 ± 3.62 MPa; r^2^ = 0.107, *P* = 0.2331; Fig. 2C).

### Changes in EHL mechanics and collagen content are dependent on feed duration

**Fig. 3:**
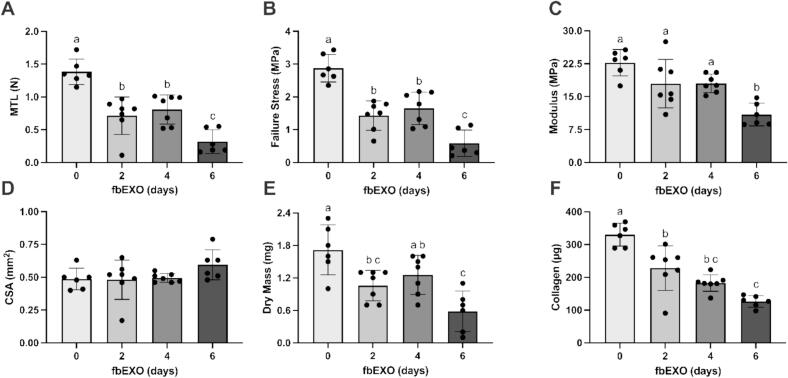
The effect of duration feeding media supplemented with 40 % FBS-derived exosome on EHL mechanical properties and collagen content. Controls are fed standard growth media containing 10 % FBS and 5 ng/ml TGF-β1. Each dot represents a technical replicate within the experimental group. Values are mean ± SD. Data were analyzed by a one-way ANOVA. A Tukey’s post hoc test was used when significant differences were identified. Matching letter pairs above each column denotes non-significant difference P ≥ 0.05.

Incubating EHLs with 40 % fbEXO for 3 feeds (6 days) resulted in EHLs with lower MTL (FBS: 1.383 ± 0.195 N, 1 feed: 0.714 ± 0.285 N, 2 feeds: 0.809 ± 0.224 N, 3 feeds: 0.318 ± 0.180 N; *P* = 0.0043; Fig. 3A), failure stress (FBS: 2.875 ± 0.418 MPa, 1 feed: 1.429 ± 0.445 MPa, 2 feed: 1.651 ± 0.498 MPa, 3 feed: 0.587 ± 0.403 MPa; *P* = 0.0015; Fig. 3B), Young’s modulus (FBS: 22.710 ± 3.027 MPa, 1 feed: 17.950 ± 5.534 MPa, 2 feed: 18.020 ± 2.076 MPa, 3 feed: 10.900 ± 2.587 MPa; *P* = 0.0049; Fig. 3C), dry mass (FBS: 1.72 ± 0.46 mg, 1 feed: 1.06 ± 0.28 mg, 2 feeds: 1.26 ± 0.36 mg, 3 feeds: 0.58 ± 0.38 mg; *P* = 0.0076; Fig. 3E), and collagen content (FBS: 330.3 ± 34.6 µg, 1 feed: 496.7 ± 159.1 µg, 2 feeds: 390.1 ± 59.4 µg, 3 feeds: 257.9 ± 42.1 µg; *P* = 0.0026; Fig. 3F) compared to 1 feed (2 days), 2 feeds (4 days) or FBS control, with no differences observed between 1 feed (2 days) or 2 feeds (4 days). No differences were observed in EHL area (FBS: 0.487 ± 0.083 mm^2^, 1 feed: 0.481 ± 0.150 mm^2^, 2 feed: 0.494 ± 0.034 mm^2^, 3 feed: 0.595 ± 0.114 mm^2^; *P* = 0.1657; Fig. 3D).

### Human Serum-Derived exosomes support EHL viability but yield structurally weaker constructs compared to FBS

**Fig. 4:**
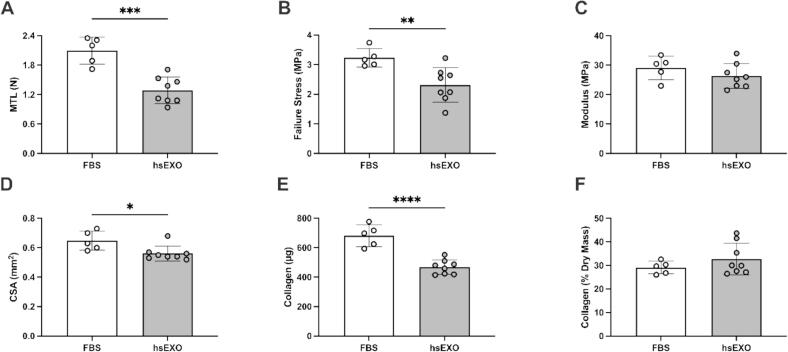
The effect of supplementing media with FBS or human serum-enriched exosomes (hsEXO) on EHL mechanical and material properties and collagen content. Values are mean ± SD. Data were analyzed by an unpaired student’s *t*-test. * Denotes significant difference.

Treating EHLs with 30 % hsEXO for 4 days (2 feeds) significantly decreased MTL (1.288 ± 0.2701 N vs. 2.094 ± 0.2761, *P* = 0.0003), failure stress (2.314 ± 0.5816 vs. 3.232 ± 0.3112, *P =* 0.0082; Fig. 4A), CSA (0.5613 ± 0.0506 vs. 0.6480 ± 0.0646 mm^2^, *P =* 0.0201; Fig. 4D), and absolute collagen content (468.5 ± 48.91 vs. 682.0 ± 73.41 µg, *P <* 0.0001; Fig. 4E), with no differences in modulus (26.32 ± 4.22 vs. 29.10 ± 3.98, *P =* 0.2632; Fig. 4C) and relative collagen content (32.71 ± 6.72 vs. 29.09 ± 2.69 %, *P =* 0.2807; Fig. 4F) when compared to treating EHLs with 10 % FBS.

### Post-Exercise hsEXO leads to similar development of EHLs compared to resting hsEXO

**Fig. 5:**
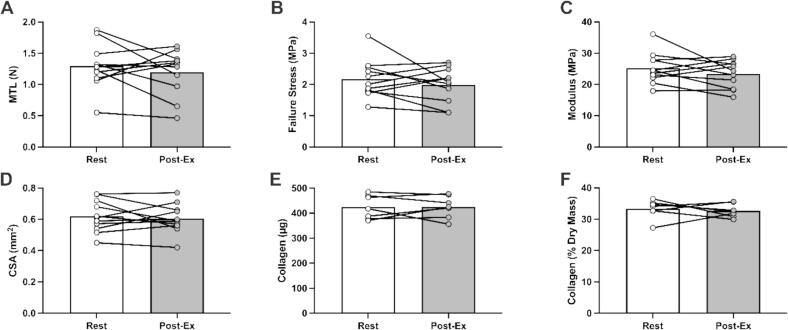
The effect of supplementing construct media with exosomes isolated from human serum obtained at rest or 15-min post-resistance exercise on EHL mechanical and material properties and collagen content. Each dot represents the average of at least two replicates for a single participant. Values from EHLs treated with hsEXOs from the same participant (before and after resistance exercise) are connected by a line. Bars represent the mean values for each group. Data were analyzed by a paired student’s *t*-test.

No significant differences were observed when EHLs were treated with hsEXO isolated from serum obtained at rest or 15 min after resistance exercise (Post-Ex) in maximum tensile load (1.30 ± 0.36 vs. 1.20 ± 0.36 N, *P* = 0.3950; Fig. 5A), failure stress (2.17 ± 0.61 vs. 1.99 ± 0.56 MPa, *P* = 0.3504; Fig. 5B), modulus (25.18 ± 4.92 vs. 23.40 ± 4.39 MPa, *P* = 0.2465; Fig. 5C), cross-sectional area (0.62 ± 0.10 vs. 0.61 ± 0.09 mm^2^, *P* = 0.5889; Fig. 5D), or absolute (424.6 ± 47.68 vs. 425.2 ± 44.46 µg, *P* = 0.9663; Fig. 5E) or relative (33.29 ± 2.99 vs. 32.70 ± 2.18 %, *P* = 0.6837; Fig. 5F) collagen content.

## Discussion

Previous work has shown that factors circulating after resistance exercise improve EHL mechanics and collagen content [5]. To determine whether exosomes play a role in this tissue crosstalk, we had to modify our EHL model to replace fetal bovine serum and TGF-β1 with exosome-enriched serum. To accomplish this, we first established the dose–response relationship between exosomes enriched from fetal bovine serum (FBS) and the mechanical properties and collagen content of EHLs. We then determined how long we could treat EHLs with fbEXO without impairing the mechanics and collagen content of the tissue. Last, we compared FBS with exosomes enriched from human serum and demonstrated that treating EHLs with hsEXO enriched from the equivalent of 30 % serum for 4 days resulted in tissues that were strong and viable, despite being mechanically weaker to those fed standard FBS and TGF-β1. This novel methodological approach allowed us to determine whether exosomes enriched from human serum obtained at rest or post-exercise contained factors that improved mechanics and collagen content. Contrary to our hypothesis, EHL mechanics and collagen content were not different when treated with hsEXO from rest or post-resistance exercise.

Acute exercise results in alterations in circulating concentrations of hundreds of molecules, identified as exercise factors [26]. These molecular responses are thought to play key roles in regulating homeostasis and substrate metabolism during and immediately following exercise [27]. However, the direct influence of these exercise factors on training-induced adaptations remains largely speculative and are only supported by circumstantial evidence. To conclusively demonstrate muscle–tendon crosstalk, we proposed that serum after exercise should result in the same biochemical or functional change at the cellular or tissue-level *in vitro* that is observed *in vivo* [18]. We have previously established a tendon/ligament organoid model that allowed us to show an increase in both tissue mechanics and collagen content using serum from people after resistance exercise, suggesting the presence of muscle–tendon crosstalk [5,22]. However, that previous work was unable to identify a hormonal explanation for the improved connective tissue function following resistance exercise.

To investigate the role of exosomes in the post-exercise serum induced improvements in ligament function, we enriched exosomes from human serum using a spin column-based procedure with affinity membrane binding (exoEASY, QIAGEN GmbH, Hilden, Germany). The presence of exosomes within our enriched samples was demonstrated by visual confirmation via TEM, showing particles in the correct size range (approximately 50–150 nm in diameter) and shape. The enrichment of exosomes was further reinforced by presence of common exosomal markers. HSP70 and Flotillin-1 were observed in both whole serum and exosome fractions. Their presence in both is not surprising as HSP70 can be present in free form and both HSP70 and flotillin-1 can be present in other vesicles as well. Alix, CD9, and CD63 were only detected within the enriched exosome fraction and not in whole serum. This indicates successful concentration of these exosomal markers. Furthermore, CD9 and CD63 were only detected in the resting exosome fraction and were not present in the post-exercise exosome fraction. This is in line with previous findings showing altered exosome release after exercise, like a diminished CD63 protein expression [28].

Small extracellular vesicles (EV) are released from myofibers *in vivo* [29] and are capable of mediating significant biological changes in recipient cells and tissues [30,31,32]. Human plasma-derived exosomes have been shown to promote expression of tendon related genes (Increased Scx, Col1a1, Col3, Tnmd, and DCN, and decreased mmp3) *in vitro* [33,34,35]. Additionally, treating injured rotator cuff tendons with an exosome preparation has been shown to increase cell proliferation, upregulate tenogenic markers, and improve repair and regeneration [34]. In line with these previous findings, we demonstrate that exosomes derived from FBS support EHL development, as evidenced by a dose–response relationship (Fig. 2) between exosome concentration and EHL mechanics and collagen content. These data confirm that serum-enriched exosomes are sufficient to support connective tissue development.

Even though fbEXO can sustain EHL development, longer treatment does result in decreased EHL mechanics and collagen content (Fig. 3). This reduction likely stems from the absence of essential signals normally activated by FBS and/or TGF-β1. In complete media, EHLs contain almost no collagen and are mechanically very weak 8 days following formation [21]. From 8 until 14 days, collagen content and EHL mechanics increase linearly [21], therefore this is an extremely anabolic stage where signals from FBS and TGF-β1 are likely essential. Notably, when feeding with FBS-enriched exosomes was limited to 2 or 4 days (started at day 10 or 12 of development) mechanics and collagen content were about half that of FBS and TGFβ1 treated controls, with no differences between those two feeding durations. Importantly, fbEXO treatment for 4 days reproducibly produced EHLs with MTLs of 0.75 N and collagen contents of ∼ 250 µg. Tissues of this strength are well suited to screen for compounds that increase, or decrease tendon or ligament structure and function [22,36,37]. We then treated EHLs with human serum-enriched exosomes for 4 days with 30 % hsEXO (to decrease the serum required from each participant) and confirmed that EHLs developed sufficiently to allow for measurement of matrix content and mechanical properties, indicating functional tissue formation (see Fig. 4). These hsEXO treated EHLs developed similarly to the fbEXO treated EHLs. The mechanics and collagen content of the newly developed EHLs were lower than FBS and TGF-β1 treated controls, but still reproducibly strong enough for investigating the impact of blood-derived exosomes. This newly established method allowed us to determine whether exosomes played a role in post-exercise muscle–tendon crosstalk using only ∼ 2.5 mL of human serum (see Fig. 5).

Post-exercise EVs exert beneficial effects in recipient cells and organs beyond skeletal muscle [38,39,40]. Additionally, exercise training influences the miRNA profile of preparations of exosomes from resting samples [41]. Nevertheless, studies on the exercise-mediated effects of EVs on connective tissue adaptations are scarce. Thus, using the model established in the current study, we sought to investigate the role of exosomes in the long-term adaptations of tendons and ligaments [[11], [12],^13^] to chronic loading. To this end, we enriched exosomes within human serum, obtained at rest and 15 min post-resistance exercise, and compared their effect on EHL structure and function. Contrary to our hypothesis, exosomes enriched from post-exercise serum created EHLs with similar mechanics and collagen content to hsEXO enriched from resting serum. This suggests that in the acute post-exercise environment the primary drivers of tendon adaptation are not exosome-based.

There were a number of limitations to the current study. First, even though we did not detect a difference in EHLs treated with hsEXO 15-minutes post exercise, it is still possible that exosomes are involved in long-term adaptations of tendons and ligaments. We only tested serum drawn 15 min after an acute resistance exercise bout. Though we have previously shown that this results in stronger EHLs with more collagen [5], this might not be the optimal time-point to collect blood. Since exerkines are released at varying time courses, it is currently unclear whether exosomes obtained from different timepoints would have different effects on the engineered ligaments. It is also possible that differences between the participants (strength training history, exercise intensity, etc.) or the training duration/equipment in the current study and the previous work resulted in a blunting of the production of exosomes. Second, as previously discussed, multiple studies have shown that skeletal muscle releases bioactive molecules and exosomes into the circulation during and after exercise [18,19]. These circulating vesicles and myokines can mediate systemic signaling, suggesting that muscle-derived signals are likely included within the serum-derived exosome population. However, we acknowledge that locally released or tissue-retained factors may differ in composition or potency, and this remains a limitation of using serum as a proxy for muscle–tendon crosstalk.

In conclusion, we demonstrate that exosomes enriched from fetal bovine serum can replace the need for FBS and TGFβ1 and support *in vitro* tendon/ligament development. Further, exosomes enriched from human serum support EHL development to the same extent as fbEXO. However, hsEXO obtained acutely after resistance exercise did not alter the mechanical properties and collagen content of EHLs when compared to hsEXO obtained at rest. Future studies can use this technique to determine the role of exosomes in connective tissue development and adaptation resulting from inactivity, disease, nutrition, etc. Last, the search continues for the factors that circulate after resistance exercise and improve tendon collagen content and mechanics.

## CRediT authorship contribution statement

**Kevin J.M. Paulussen:** Methodology, Formal analysis, Investigation, Data curation, Project administration, Visualization, Writing – original draft. **Christopher M.T. Hayden:** Formal analysis, Investigation, Methodology, Writing – review & editing. **Taylor Griffin:** Writing – review & editing, Methodology, Formal analysis. **Keith Baar:** Writing – review & editing, Supervision, Resources, Project administration, Investigation, Funding acquisition, Data curation, Conceptualization.

## Declaration of competing interest

The authors declare the following financial interests/personal relationships which may be considered as potential competing interests: Keith Baar reports financial support was provided by Joe and Clara Tsai Foundation.

## Data Availability

Data will be made available on request.
